# The influence of the Great Recession on perinatal health—an ecological study on the trend changes and regional differences in Portugal

**DOI:** 10.1016/j.lanepe.2023.100735

**Published:** 2023-09-08

**Authors:** Julia Nadine Doetsch, Ricardo Almendra, Milton Severo, Teresa Leão, Raquel Teixeira, Sandra Marques, Eva Pilot, Thomas Krafft, Henrique Barros

**Affiliations:** a EPIUnit – Instituto de Saúde Pública da Universidade do Porto, Portugal; b Laboratório para a Investigação Integrativa e Translacional em Saúde Populacional (ITR), Porto, Portugal; c Faculty of Health, Medicine and Life Sciences, Care and Public Health Research Institute, Maastricht University, Maastricht, the Netherlands; d Department of Geography and Tourism, CEGOT-Centre of Studies on Geography and Spatial Planning, University of Coimbra, Portugal; e Instituto de Ciências Biomédicas Abel Salazar, Universidade do Porto, Portugal; f Faculdade de Medicina, Departamento de Ciências da Saúde Pública e Forenses e Educação Médica, Universidade do Porto (FMUP), Porto, Portugal; g ICNOVA, FCSH, Universidade Nova de Lisboa, Portugal

**Keywords:** Great Recession, Infant mortality, Perinatal mortality, Socioeconomic factors, Mortality trends

## Abstract

**Background:**

Few studies examine the relationship between socioeconomic factors and trends in mortality in high-income European countries. Due to the lack of regional-level data, most recent studies on social inequality in Portugal do not investigate regional differences. This study analyses time trends and regional disparities in the evolution of perinatal mortality (PMR) and infant mortality (IMR) associated with demographic and socioeconomic indicators following Portugal's 2008 economic and financial crisis.

**Methods:**

Associations were assessed using generalised linear models. A Poisson joinpoint regression model was applied to identify relevant PMR and IMR changes between 2000 and 2018. Country regional disparities were analysed using Mixed Effect Multilevel models.

**Findings:**

IMR and PMR significantly decreased in the pre-crisis period but not in the post-crisis period. The significant differences between regions in IMR and PMR in 2000 were followed by a different evolution of regional IMR after 2008. PMR and IMR were not significantly associated with socioeconomic indicators. A significant positive association with maternal age at first birth was identified.

**Interpretation:**

Results confirm the influence of the crisis on PMR and IMR trends in Portugal, taking into account recurring associations between macroeconomic cycles, variations in mortality trends, macroeconomic volatility, and stagnation of IMR and PMR. Regional inequalities confirm the internal variability of the crisis influence and persistent spatial inequalities affecting IMR patterns.

**Funding:**

10.13039/501100019370FCT, under the Institute of Public Health of the 10.13039/501100006752University of Porto (ISPUP)–EPIUnit (UIDB/04750/2020) and ITR (LA/P/0064/2020), 10.13039/501100001835Maastricht University’s external PhD programme under the Care and Public Health Research Institute (10.13039/501100011095CAPHRI), and the RECAP preterm project (grant agreement no 733280).

**Research in context**

**Evidence before this study**

Socioeconomic factors appear to be less important in elucidating perinatal and child health during periods of economic growth in high-income countries but it is unknown whether that is also verified in times of crises. It is still controversial whether income is causal in determining perinatal health in low- and middle-income countries. Very few studies examined the association between socioeconomic factors and perinatal and infant mortality trends in high-income countries in Europe, and to our knowledge none yet in Portugal. We accessed online open-access annual perinatal, demographic, and socioeconomic data at NUTS II level collected by Eurostat and Statistics Portugal for the period 2000–2018 in Portugal. We selected the indicators: Infant mortality rate (‰), Perinatal mortality rate (‰), Maternal age at first birth (years), Unemployment rate (total annual) (%), Long-term unemployment rate (total annual) (%), Gross domestic product (GDP) (%), and Gross household disposable income per inhabitant (base 2016–1000€).

**Added value of this study**

Evidence indicated the negative influence of the 2008/2009 financial and economic crisis on perinatal and infant mortality trends in Portugal, mortality rates slowed down after 2008/2009. Infant and perinatal mortality trends differed statistically among the Portuguese NUTS II regions. Maternal age at first birth plays a decisive role in perinatal mortality in Portugal.

**Implications of all the available evidence**

Results stress the importance of taking the recurring association between macroeconomic cycles, variations in mortality trends, macroeconomic volatility, and stagnation of mortality trends into account when analysing the 2008/2009 economic and financial crisis. Identified regional disparities in infant mortality require actions in mother-and-child healthcare interventions at the local level suitable especially to the unemployed, urging action for decentralization. Results argue for the relevance of considering the political economy of global health finance and macroeconomic volatility as a major influence on the association of perinatal and infant mortality rates with socioeconomic indicators highlighting their importance for the policymaking process. Mortality patterns refer to the internal variability of the crisis’ effect and to persistent spatial regional inequalities appealing to the need for attention in policymaking.

## Introduction

The 2008/2009 economic and financial crisis, also called the Great Recession, caused the largest decline in gross domestic product (GDP), income decline, and increases in unemployment and long-term unemployment rates for large parts of the population in the post-war era.^1^ With the rapid drop in real GDP (5.3% for Q4 2010–Q4 2012) and implemented austerity measures, the Portuguese unemployment rate has increased as one of the fastest rates in the European Union (EU) (16% in 2012)^2^ affecting particularly young people.^3^ Income distribution, activity status, and education are important contributors to health inequality.^4^ Post-crisis and associated macroeconomic policy effects (e.g., fiscal policy) have been discussed to have deteriorated the quality of perinatal care for very preterm (<32 weeks of gestation),^5^ low-birthweight births (≤2500 g),^6^ increased child poverty,^3^ and amplified inequalities in various EU countries (e.g., Portugal, Greece, Italy and Spain).^7^

The degree of health inequalities and social grade can be assessed through the infant mortality rate (IMR) as a robust indicator of population health and health systems' effectiveness.^8^ An important indicator of the health condition of a country is the perinatal mortality rate (PMR). PMR is also a sensitive marker of the quality of health care provided during pregnancy, delivery and the early postpartum period.^5^ These indicators are substantially impacted by economic factors such as income and socioeconomic development.^9^ Therefore, enhancements in economic conditions are a driving force behind its magnitude which was reflected in EU accession candidate countries,^10^ and in high-income countries.^11^

However, in high-income countries, socioeconomic factors seem to be less critical in explaining PMR and IMR in times of economic growth.^12^ Socioeconomic factors might be able to buffer the adverse outcomes of an economic crisis. Yet, it is debatable whether that is verified in times of crisis.^13^ Previous international studies associated economic downturns with increases in IMR reported a negative association between per capita (GDP) and IMR, and debated the role that income plays in determining perinatal health in low- and middle-income countries but continue to be controversial.^14^^,^^15^ Although some international studies have examined this association in high-income European countries,^16^^,^^17^ up to now, this has hardly been specifically examined in Europe, particularly in those countries with economies very exposed to external shocks, such as Portugal.^13^

Portugal has one of the most unequal income distributions in Europe, and poverty levels are high.^18^ The largest regional disparities are found in health and access to services.^18^ On access to services, Lisbon ranks among the best 35% of OECD regions and Alentejo among the worst 10%.^19^ Moreover, Rodrigues (2019) argues that the economic crisis had a greater influence on urban areas in Portugal.^19^ However, most recent studies on poverty, social exclusion and social inequality in Portugal do not examine regional differences due to a lack of data at the regional level.^19^

We hypothesise that time trends and regional disparities in the evolution of PMR and IMR are associated with demographic and socioeconomic indicators following Portugal's 2008 economic and financial crisis. We tested this hypothesis by analysing the trend changes and evaluating regional disparities in the evolution of PMR and IMR associated with GDP, household income, unemployment, long-term unemployment rate, and maternal age at first birth.

## Methods

### Study area

Socioeconomic inequalities are present across Portuguese NUTS II regions (Supplementary Table S1). NUTS stands for “Nomenclature of territorial units for statistics” and is a hierarchical system for dividing up the economic territory of the European Union defined by EUROSTAT. Portugal is located in Western Europe and is constituted by 7 NUTS II (North, Centre, Lisbon metropolitan area, Alentejo and Algarve in the mainland and two Azores and Madeira islands). Portugal has nearly 10 million inhabitants, of which the majority live in the coastal area and the two most populous cities, Lisbon and Oporto which account for 4.5 million inhabitants.

### Study design

This analysis is an ecological study that uses longitudinal data from the period 2000–2018. Data were assessed at the national and regional levels to analyse potential regional disparities. We defined the end-2008/beginning-2009 as the year of change when the global financial crisis of 2007–2008 hit Europe, including Portugal, according to Kana et al. (2017) and Zilidis et al. (2020).^6^^,^^13^ Therefore, we defined 2000–2008 as the pre-crisis period and 2009–2018 as the post-crisis period.

### Data

Perinatal, demographic and socioeconomic yearly data were collected from Eurostat and Statistics Portugal (INE) (Table 1):

(i) **Infant mortality rate** (IMR) is assessed by the number of infant deaths before reaching the first year of life per 1000 live births (%);
(ii) **Perinatal mortality rate** (PMR) is calculated as the number of perinatal deaths stillbirths and newborn deaths before reaching the first 7 days of life per 1000 total births (stillbirths and live births) (%);
(iii) **Maternal age at first birth** (years) refers to the age of the mother when she gave birth to her first child;
(iv) **Unemployment rate (total annual)** (%) is an indicator of economic and social well-being. It is defined as the number of unemployed persons as a percentage of the labour force;
(v) **Long-term unemployment rate (total annual)** (%) shows the proportion of long-term unemployed (for 12 months or more) among all unemployed;
(vi) **Gross domestic product (GDP)** per inhabitant in purchasing power parity (%) (EU28 = 100) is a measurement of the wealth within an economy. GDP is defined as the level of output that an economy can produce at a constant inflation rate;
(vii) **Gross household disposable income** per inhabitant (base 2016–1000€) (NUTS–2013) is the income available to households such as wages and salaries, income from self-employment and unincorporated enterprises, income from pensions and other social benefits, and income from financial investments.

**Table 1:** Indicators and data sources.

Dimension	Indicator	Unit of measure	Source
Perinatal	Infant mortality rate	Per thousand (‰)	Statistics Portugal
	Perinatal mortality rate	Per thousand (‰)	Statistics Portugal
Demographic	Maternal age at first birth	years	Eurostat
Socioeconomic indicators	Unemployment rate	percentage	Eurostat
	Long-term unemployment rate	percentage	Eurostat
	Gross domestic product (GDP)	percentage	Statistics Portugal
	Gross household disposable income	1000 Euros	Statistics Portugal

Authors JD and RA accessed and verified the data.

### Patient and public involvement

None.

### Statistical analysis

A range of rigorous statistical models are applied in this work. The level of significance was set at p < 0.05; model estimates and respective high and low Confidence Intervals (CI) are reported.

#### Association between PMR and IMR with GDP

The associations between PMR and IMR with GDP, household income, unemployment, long-term unemployment rate and maternal age at first birth were assessed through separated generalised linear models with a Gaussian distribution, adjusted by time.

#### Time trend analysis

We applied a Poisson joinpoint regression model to explore time trend changes in PMR and IMR following the Great Recession. We analysed the period from 2000 to 2018. We set the cut-point at the end of 2008 to assess if the Great Recession had a significant outcome on IMR and PMR, corresponding to previous studies.^6^^,^^13^ We estimated Annual Percent Change (APC) and the Average Annual Percent Change (AAPC) for PMR and IMR, based on the best fitting model, in each period. Time trend analyses were performed with the Joinpoint Regression Program (version 4.9.1.0 April 2020).

#### Regional differences

Mixed Effect Multilevel models with an interrupted time series structure were applied to analyse regional disparities of the influence of the 2008 economic and financial crisis on PMR and IMR in Portugal across NUTS II regions. Models with random intercept were compared with models with random intercept and random slope through ANOVA. In the random intercept models, the intercept can vary between regions while taking different starting points for infant and perinatal indicators into account. In addition, the random intercept and random slope models can also capture different trends across regions. Thus, Gaussian models included terms for time (measured in units of 2 years), time since 2008 ((time-2008)_+_; also modelled in units of 2 years) and a dummy variable splitting the time series in two (before and after 2008) were built. The ANOVA comparison allows us to evaluate which of the presented hypotheses better represents the evolution of perinatal indicators across regions.

Furthermore, in case of signs of regional disparities in the evolution of the health outcomes under analysis (through ANOVA) an interaction term between time since 2008 and the scaled socioeconomic variables (as z-scores) was added to evaluate if regional disparities in the evolution of IMR and PMR after the crises were associated with socioeconomic inequalities.

Models evaluating regional differences were developed using R software version 4.0.2 (R Core Team, 2019).

### Role of the funding source

The salary of JD was paid during the initial phase of the study (creating the **“Study design”** and **“Data collection”**) by the RECAP preterm project and has received funding from the European Union’s Horizon 2020 research and innovation programme under the grant agreement No 733280. The salary of JD during the **“Writing process of the report”**, **“Data analysis”** and **“Interpretation”** was paid by the Foundation for Science and Technology—FCT (Portuguese Ministry of Science, Technology and Higher Education), under the Unidade de Investigação em Epidemiologia—Instituto de Saúde Pública da Universidade do Porto (EPIUnit) and the Laboratório para a Investigação Integrativa e Translacional em Saúde Populacional (ITR) UIDB/04750/2020 and LA/P/0064/2020. The **“Publication of the study”** was funded by the external PhD programme of Maastricht University, Faculty of Health, Medicine and Life Sciences (FHML), Care and Public Health Research Institute (CAPHRI), the Netherlands, that JD is enrolled in.

The sponsors had no involvement in the **“interpretation of the findings”** of the study.

## Results

### National analysis

From 2000 to 2008 (pre-crisis) and 2009 to 2018 (post-crisis), different trends in PMR and IMR were observed. The average rate of IMR and PMR decreased during both periods. A significant AAPC decrease was observed for IMR (−6.6%) and PMR (−5.1%), in the pre-crisis period but not for the post-crisis period (−0.8%; −1.4%, respectively) (Table 2).

**Table 2:** Average Annual Percent Change within the periods of 2000–2008, 2009–2018 in Portugal.

	Infant mortality rate	Perinatal mortality rate
Average rate		
Average rate		
2000–2008	4.10	4.95
2009–2018	3.05	3.85
Average annual percent change (%) (CI)		
2000–2008	−6.6 (−9.7 to −3.5)	−5.1 (−8.4 to −1.5)
2009–2018	−0.8 (−2.5 to 0.8)	−1.4 (−2.9 to 0.1)
Test statistic (t-test)		
2000–2008	−4.1	−2.8
2009–2018	−1.1	−2.1
p-value		
2000–2008	**<0.001**	**<0.001**
2009–2018	0.3	0.1

Bold text indicates significant results (<0.005). CI, confidence interval.

There were significant positive associations between PMR and IMR with maternal age at first birth. The associations between PMR and IMR with GDP, household income, unemployment rate and the long-term unemployment rate were non-significant. Despite the non-significance, there were positive associations for PMR with household income and an inverse association with GDP, unemployment and long-term unemployment. For IMR, there were inverse associations with GDP, household income, unemployment and long-term unemployment (Table 3).

**Table 3:** Association^a^ between perinatal and infant mortality with GDP, household income, unemployment rate, long-term unemployment rate and maternal age at first birth, in Portugal.

Socioeconomic indicators	Infant mortality	Perinatal mortality
GDP		
Estimates (CI)	−0.030 (−0.067 to 0.008)	−0.009 (−0.042 to 0.024)
SE	0.019	0.017
p-value	0.143	0.588
Household income		
Estimates	−0.027 (−0.151 to 0.09)	0.002 (−0.093 to 0.093)
Standard error (SE)	0.058	0.045
p-value	0.646	0.959
Unemployment		
Estimates	−0.024 (−0.051 to 0.004)	−0.018 (−0.038 to 0.003)
SE	0.013	0.010
p-value	0.091	0.110
Long-term unemployment		
Estimates	−0.037 (−0.079 to 0.007)	−0.028 (−0.061 to 0.005)
SE	0.021	0.016
p-value	0.099	0.106
Maternal age at first birth		
Estimates	0.666 (0.207–1.141)	0.428 (0.034–0.832)
SE	0.235	0.201
p-value	**0.012**	**0.049**

Bold text indicates significant results (<0.005). SE, standard error.

a Association measured through separated generalised linear models with a Gaussian distribution, adjusted by time.

### Regional analysis

There were significant differences in IMR and PMR between the regions at the beginning of the period under study (2000) (Table 4).

**Table 4:** Mixed effect model for regional disparities in perinatal and infant mortality trend 2000–2018 by NUT regions, in Portugal.

	Infant mortality			Perinatal mortality		
	Estimates	CI	p	Estimates	CI	p
Intercept	1.739	1.561–1.916	**<0.001**	1.852	1.738–1.965	**<0.001**
Time^a^	−0.151	−0.212 to −0.090	**<0.001**	−0.088	−0.127 to −0.050	**<0.001**
Time since 2008^b^	0.122	0.045–0.199	**0.002**	0.053	−0.001 to 0.107	**0.050**
Before 2008	Ref					
After 2008	0.081	−0.071 to 0.232	0.294	0.020	−0.138 to 0.178	0.802
Random effects standard deviation						
Intercept	0.181			0.114		
Time^a^	0.054			–		
Time since 2008^b^	0.065			–		
Residual	0.814			1.105		

Bold text indicates significant results (<0.005).

a Time measured at a 2 years unit.

b Time Since 2008 measured at a 2 years unit.

**Fig. 1:**
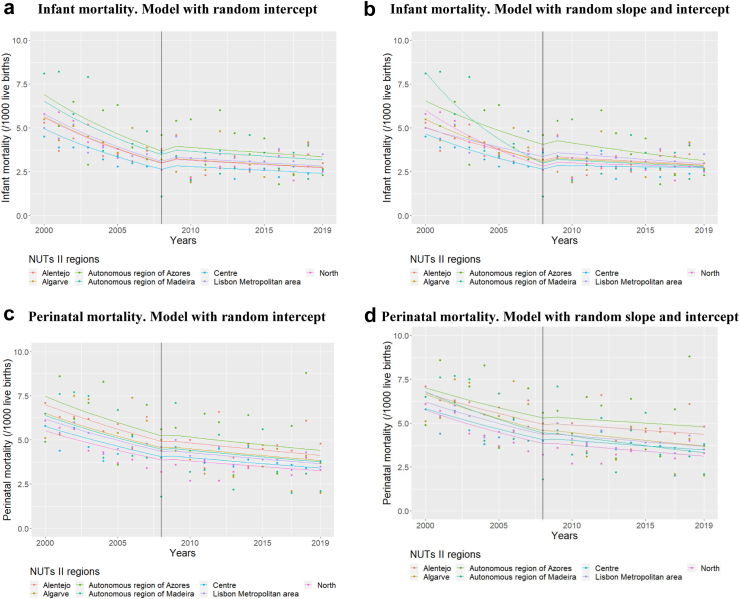
**Interrupted time series with level change regression model for infant and perinatal mortality at regional level (NUTS II) inPortugal.**

The ANOVA results indicate that the model accounting for random slope and random intercept is more adequate to explain the evolution of IMR across regions (Anova p-value: 0.004) as different decreasing trends of IMR, both before and after the crisis, were found. The evolution of PMR is more constant as no such regional difference was found (Anova p-value: 0.654) (Table 4 and Fig. 1).

Considering that IMR evolution is significantly different across regions, we tested if such differences were associated with socioeconomic inequalities by adding an interaction term to the model. No evidence was found of the considered demographic and socioeconomic indicators contributing to the observed trends (Table 5).

**Table 5:** Regional disparities in the evolution of infant mortality associated with socioeconomic indicators after the Great Recession in 2000–2018 in Portugal.

	GDP			Household income			Long term unemployment			Unemployment			Maternal age at first birth		
	β	CI	p-value	β	CI	p-value	β	CI	p-value	β	CI	p-value	β	CI	p-value
Intercept	1.741	1.567–1.915	**<0.001**	1.766	1.528–2.003	**<0.001**	1.600	1.316–1.884	**<0.001**	1.568	1.318–1.819	**<0.001**	1.721	1.548–1.893	**<0.001**
Time^a^	−0.151	−0.211 to−0.091	**<0.001**	−0.159	−0.239 to−0.079	**<0.001**	−0.112	−0.179 to−0.045	**0.001**	−0.104	−0.193 to−0.014	**0.024**	−0.138	−0.199 to−0.077	**<0.001**
Time since 2008^b^	0.123	0.043–0.203	**0.003**	0.116	0.011–0.222	**0.031**	0.102	0.027–0.177	**0.008**	0.080	−0.034 to0.193	0.168	0.111	0.035–0.187	**0.005**
Before 2008	Ref			Ref			Ref			Ref					
After 2008	0.079	−0.072 to0.230	0.303	0.094	−0.075 to0.263	0.275	−0.014	−0.192 to0.164	0.877	0.041	−0.145 to0.227	0.662	0.036	−0.123 to0.196	0.653
Socioeconomic indicator	−0.019	−0.123 to0.084	0.710	0.020	−0.106 to0.145	0.756	−0.067	−0.214 to0.079	0.364	−0.016	−0.159 to0.128	0.829	0.034	−0.009 to0.078	0.117
Interaction time since 2008^b^ ∗socioeconomic indicator	0.023	−0.003 to0.049	0.087	0.015	−0.019 to0.050	0.379	0.034	−0.013 to0.081	0.151	0.012	−0.037 to0.061	0.623	−0.012	−0.033 to0.010	0.281

Bold text indicates significant results (<0.005). ∗, interaction with.

a Time measured at a 2 years unit.

b Time Since 2008 measured at a 2 years unit.

## Discussion

IMR and PMR have been decreasing during the study period. They have decreased at a significant AAPC in the pre-crisis period (2000–2008) but not in the post-crisis period (2009–2018). PMR and IMR were significantly positively associated with maternal age at first birth but not with any of the national-level socioeconomic indicators. Across regions, significant differences were also found in the evolution of IMR and PMR until 2008, but after 2008 only for IMR.

### PMR and IMR trends

The decreasing trend of PMR and IMR and the significant differences between the pre and post-crisis periods mirror the outcome of the 2008 crisis, as reported in other European countries.^20^ Results may further reveal the recurring association between macroeconomic cycles and variations in mortality trends.^21^ In Portugal, IMR and PMR have presented a long decreasing trend over the last five decades, similar to most European countries.^13^ This decreasing trend has been associated with overall improvements in healthcare (e.g., vertical mother-child health programs)^22^ and economic and social transformations (e.g., housing) in Portugal.^23^ A stagnation in IMR may also explain the slower decrease of IMR since 2008 when compared with previous decades.^24^

### Association PMR and IMR trends with socioeconomic indicators

The associations between PMR and IMR with maternal age at first birth, measured through a Gaussian model adjusted by time, were statistically significant, as previous studies revealed in Portugal and elsewhere.^25^^,^^26^ Despite the non-significance, there were inverse associations for PMR with GDP, unemployment and long-term unemployment; for IMR, there were inverse associations with GDP, household income, unemployment and long-term unemployment. According to De Curtis (2014), the recent economic recession has worsened social conditions and further increased unemployment.^27^ Similarly, our results, although non-significant, may also suggest a potential counter-cyclical influence.^27^ In Portugal, crisis-induced modifications in the labour market structure led to an increase in income inequality, material deprivation and unemployment (7.7% in 2008 to 15.9% in 2012 and 16.7% in 2013) affecting one-fifth of households.^28^ Consequently, the overall increased risk of poverty during crisis events, particularly of children (23.0% in 2010; 26.8% in 2012; 24.2% in 2017) was reported.^6^^,^^29^

As previous studies reveal, socioeconomic conditions play a decisive role in perinatal outcomes,^20^ and their changes highly affect PMR and IMR, associating a higher risk of poverty with higher IMR.^13^ In Portugal, a previous study reported higher mortality experienced by most deprived groups at birth between 2010 and 2012.^30^ The crisis-induced deceleration in GDP and the decrease in health expenditure and social protection distribution on family/children support overlapped with increasing low-birthweight risk (<2.500 g) in Portugal, as also described in other European countries (e.g., Italy, Greece, Iceland).^6^^,^^28^

The interpretation of the role of socioeconomic data with mortality rates must be conducted carefully, thus considering a possible pro-cyclical or counter-cyclical relationship.^31^ The previously discussed general downward trend in IMR and PMR, changes in the social profile and health behaviours of childbearing women, medical advances, and policies must be taken into consideration, once the observed decrease may be related to a long-term mortality trend.^31^ For instance, the policy “National Program of Maternal and Neonatal Health”, implemented in 2006, contributed to a decreasing IMR trend by closing small maternity units.^22^ Hence, the non-significant but positive association between PMR with GDP and household income, may potentially be linked with either a pro-cyclical influence^32^ or may potentially reflect the result of conjugation of a long decreasing trend in PMR with decreases in GDP and household income, verified after 2008.

Hence, macroeconomic volatility and the period under analysis may have influenced the results. Socioeconomic indicators vary more volatile—the liability to change rapidly and unpredictably especially for the worse—compared to PMR and IMR.^33^ Thus, it is crucial to consider the political economy of global health finance and to include debt aid as a crisis consequence. In this regard, total macroeconomic volatility, as a major contributor to our results, can be reasoned to be mainly influenced by debt aid (e.g., troika bailout packages),^34^ as applied in the Portuguese health system in response to the crisis through the troika agreement (2011–2014).

In Portugal, debt aid associated with austerity measures implied lean government involvement and economic liberalization policies to stimulate the private sector.^5^ When evaluating the consequences of volatility for respective health systems, health system spending grew at about half the speed in troika-borrowing countries (e.g., Portugal) compared to non-troika-borrowing countries, presumably reflecting troika's macroeconomic policies, which explicitly encourage governments to redirect aid to reserves to manage aid volatility.^35^ Portugal, as one of the European countries that were classified as having implemented higher levels of austerity, *inter alia* reported a substantially higher increase of low-birthweight rates in particular among migrant women^28^ and deteriorated perinatal healthcare quality for very-preterm and very low-birthweight infants.

### Regional differences in IMR and PMR trends associated with socioeconomic factors

This study found significant differences between regions in IMR and PMR in 2000 and also on the evolution of IMR after 2008. Firstly, this observation confirms previously determined uneven consequences of the 2008/2009 financial and economic crisis in Portugal.^36^ Secondly, it mirrors previously identified disparities in IMR between Portuguese regions.^37^ As earlier described, Lisbon and Vale do Tejo and Alentejo regions performed worse for IMR and PMR than the Centre and North regions.^22^ These results may result from the disproportionate geographical location of healthcare facilities, particularly maternity units, and the ease of healthcare access and utilisation.^38^^,^^39^ Prevalent regional and spatial inequalities in access to maternity units have significant implications on IMR^38^ and may lead to avoidable IMR.^22^

In wealthier regions (higher GDP or higher income) and regions with higher unemployment and long-term unemployment rates, IMR tended to be higher after the crisis. These results confirm substantial internal variability across NUTS II regions.^36^ In 2011, the Northern and Alentejo regions presented similar resilience, the Centre and Lisbon and Vale do Tejo regions presented stronger employment resilience, and the Algarve region showed a much higher level of GDP resilience compared with the national average, potentially explained mainly due to the influence of the tourism industry.^36^

We further argue that the influence of the crisis may have been felt to a stronger extent in the regions where income is higher. This is in line with an OECD report that disclosed that the percentage of the annual average change in real disposable household income decreased to a higher extent in the top decile income group (−3.7%) within the defined post-crisis period (2007–2012) when compared to the bottom decile income group (−1.9%).^40^ Greater income decrease may influence IMR to a greater extent. This corresponds to previous studies revealing a robust relationship between shocks to per capita GDP and IMR.^32^

Higher unemployment is related to higher rates of IMR and worse birth outcomes, possibly due to stress-related endocrine system changes.^16^ Previous studies further confirmed that mothers with inadequate prenatal care or with medical risk factors had significantly poorer birth outcomes when they were unemployed compared to when they were employed.^16^ This consequence of short-term economic downturns, measured as rises in unemployment rates, and their association with increases in IMR, is supported by Leahy, Healy, and Murphy (2013).^29^ These authors argue that a fair solution to a debt crisis must be found requesting alternatives for policies prioritising austerity and integrating economic and social policies.^29^

### Limitations

The ecological nature of this study imposes inherent limitations. Firstly, the study design does not allow the identification of the mechanisms leading to the adverse effects; thus, causality is out of the scope of the study design. Secondly, the results are based on the time trend of aggregated data. This implies that there are no data on the causes of the individual deaths in order to directly relate the deaths to the consequences of the 2008/2009 economic and financial crisis. In addition, the study of this phenomenon would benefit from the inclusion of further socio-economic indicators, such as maternal education, however, data were not available at the NUTS II level. This stresses the importance of data availability and data harmonization to foster scientific research.^19^^,^^41^^,^^42^

### Final remarks

Assessing the influence of the financial crisis on the healthcare system and overall public health is challenging in the interim, as most epidemiological data, including morbidity and mortality rates, have a latency period of 2–5 years.^43^ The consequences of the crisis may have been felt at different points in time across Europe and its consequences may have been delayed, as several authors argue.^13^ Even though prenatal care is accessible and remains free of charge in Portugal, it is important to ensure that economic downturns and austerity policies do not negatively affect the availability, access and use of these services,^44^ which influence IMR and PMR patterns. Although public prenatal and infant care may attenuate the consequence of economic hardship on health associated with crisis events,^4^ it only partially reduces its burden.^44^ Diversity in perinatal indicators at the regional level asks for the need for specifically tailored measures (e.g., community-based programs) to overcome these inequalities.^38^

### Conclusion

IMR and PMR have been decreasing, corresponding to the overall EU trend, but the decreasing pace significantly differed between 2000–2008 and 2009–2018. Results emphasise the influence of the crisis and may also suggest a recurring association between macroeconomic cycles, variations in mortality trends and stagnation in IMR and PMR over the last decade. At the national level, IMR and PMR were significantly associated with maternal age at first birth and non-significantly associated with socioeconomic indicators. Important regional inequalities have been found in 2000 for IMR and PMR and in the evolution of IMR after 2008. Results highlight strong internal variability across NUTS II regions in response to the crisis and prevalent regional disparities in health, which we relate to spatial inequalities affecting IMR patterns. However, when looking at summary measures, some of the relevant influences on the more vulnerable social populations may not have been revealed.

## Contributors

Julia Nadine Doetsch: Conceptualization, Methodology, Formal analysis, Investigation, Writing—Original Draft, Writing—Review & Editing, Visualization.

Ricardo Almendra: Methodology, Formal analysis, Writing—Original Draft, Writing—Review & Editing.

Milton Severo: Methodology, Formal analysis; Writing—Review & Editing.

Teresa Leão: Methodology, Writing—Review & Editing.

Raquel Teixeira: Writing—Review & Editing.

Sandra Marques: Writing—Review & Editing.

Eva Pilot: Writing—Review & Editing.

Thomas Krafft: Writing—Review & Editing.

Henrique Barros: Methodology, Writing—Review & Editing, Supervision, Project administration, Funding acquisition.

All authors/contributors were responsible for the decision to submit the manuscript.

## Data sharing statement

All data that was used is publicly available at: Eurostat and Portuguese National Statistics (INE). All data that was generated is included in this article.

## Declaration of interests

All authors declare that they have answered every question and have not altered the wording of any of the questions on the “ICMJE COI Form”.
