# Supplementary material for: The influence of the Great Recession on perinatal health—an ecological study on the trend changes and regional differences in Portugal
Source: Lancet Reg Health Eur. 2023 Sep 8;34:100735. doi: 10.1016/j.lanepe.2023.100735 (PMC10625015; doi:10.1016/j.lanepe.2023.100735)
Supplement: Supplementary Table S1 [file mmc1.docx]

**Supplementary Table 1. Demographic, socioeconomic and perinatal indicators at NUTS II level in Portugal, 2019**

| **NUT II Regions** | **GDP per capita (%)** | **Unemploy-ment (%)** | **Long-term unemploy-ment (%)** | **Gross household disposable income* (Euro)** | **IMR (per 1000)** | **PMR (per 1000)** | **Maternal age at first birth** |
| --- | --- | --- | --- | --- | --- | --- | --- |
| Northern | 67.3 | 6.7 | 3 | 11829 | 2.5 | 3.3 | 28.5 |
| Centre | 68.5 | 5 | 1.8 | 12652 | 2.3 | 3.8 | 28.6 |
| Lisbon metropolitan area | 102.1 | 7.2 | 3.1 | 15545 | 3.5 | 3.5 | 28.7 |
| Alentejo | 72.2 | 6.9 | 3 | 12919 | 3 | 4.8 | 28.9 |
| Algarve | 88 | 7.1 | 2 | 16064 | 2.7 | 2 | 29 |
| Azores | 69.9 | 7.9 | 4.1 | 13088 | 2.3 | 3.7 | 29.2 |
| Madeira | 75.8 | 7.1 | 3.7 | 12985 | 2.6 | 2.1 | 29.3 |

* latest data available was 2018

Data source: Portuguese National Statistics (INE)
